# Diatom-inspired silicification process for development of green flexible silica composite aerogels

**DOI:** 10.1038/s41598-024-57257-x

**Published:** 2024-03-23

**Authors:** Valerie Tan, Florian Berg, Hajar Maleki

**Affiliations:** 1 Department of Chemistry, Institute of Inorganic Chemistry, University of Cologne, Greinstresse 6, 50939 Cologne, Germany; 2 Center for Molecular Medicine Cologne, CMMC Research Center, Robert-Koch-Str. 21, 50931 Cologne, Germany

**Keywords:** Biomimetic, Composite aerogel, Flexible aerogel, Surface modification, Diatom-inspired silicification, Green chemistry, Inorganic chemistry, Materials chemistry, Nanoscale materials, Soft materials, Chemistry, Energy science and technology, Engineering, Materials science, Nanoscience and technology

## Abstract

In this study, we have developed novel biomimetic silica composite aerogels and cryogels for the first time, drawing inspiration from the natural diatom’s silicification process. Our biomimetic approach involved the modification of tyrosinase-mediated oxidized silk fibroin (SFO) surfaces with polyethyleneimine (PEI). This modification introduced ample amine groups onto the SF polymer, which catalyzed the silicification of the SFO-PEI gel surface with silicic acid. This process emulates the catalytic function of long-chain polyamines and silaffin proteins found in diatoms, resulting in a silica network structure on the primary SFO-PEI network gel’s surface. The SFO-PEI gel matrix played a dual role in this process: (1) It provided numerous amine functional groups that directly catalyzed the silicification of silicic acid on the porous structure’s exterior surface, without encapsulating the created silica network in the gel. (2) It served as a flexible mechanical support facilitating the creation of the silica network. As a result, the final ceramic composite exhibits a mechanically flexible nature (e.g., cyclic compressibility up to 80% strain), distinguishing it from conventional composite aerogels. By mimicking the diatom’s silicification process, we were able to simplify the development of silica-polymer composite aerogels. It eliminates the need for surfactants, multi-step procedures involving solvent exchange, and gel washing. Instead, the reaction occurs under mild conditions, streamlining the composite aerogels fabrication process.

## Introduction

Composite aerogels, consisting of inorganic and organic precursors, possess combined and synergistic properties of both material realms^1^. The most commonly studied composite aerogels are silica composite aerogels^2^, with a global market in the field of thermal insulation worth more than 250 million euros per annum and an annual growth rate of 20%^2^. These silica aerogel composites are often hybridized with polymeric organic precursors such as epoxides^3^, polyurea^4^ and polyurethane^5^ and various biopolymeric matrix including cellulose^6^, pectin^7^, alginate^8^, starch^9^, silk fibroin^10^ at molecular length scale—to name a few. The organic precursors may be physically crosslinked with the surface of silica nanoparticles in the gel network (Class I hybrid aerogel) or chemically by more robust chemical crosslinking approaches (Class II hybrid aerogel) with prior surface modification of silica network structures using tri-functional organo(alkoxy)silane having a specific functional groups in their molecular structure such as –NH_2_, –SH, –COOH, epoxide, –N_3,_ etc.^11^. While these reinforcement strategies have improved the mechanical strength and flexibility of pristine silica and silsesquioxane aerogel to several orders of magnitude, the resulted mechanical strength is still insufficient for load bearing applications^1^. Additionally, the silica components’ exact properties in the composite aerogel cannot be realised as, in most cases, the ceramic network (e.g. silica network) are encapsulated by polymeric organic phase, inevitably compromising its nanoporosity and physicochemical properties. In order to capitalise on the true potential and benefits of both ceramic and polymeric components in aerogel composites with outstanding mechanical strength and flexibility, in this study for the first time, we explored a sustainable approach to develop green silica-polymer composite aero- and cryogels with inspiration from the biosilicification process in diatoms.

Diatoms (*Bacillariophyceae*) are a large group of unicellular photosynthesizing microalgae which make up a significant portion of the Earth’s aquatic biomass^12^. Up until today, there are more than 10,000 species of diatoms^13^ forming most of the organic material in the ocean^14^, and they collectively produce 20–30% of the oxygen we breathe in Ref.^15^. The most prominent feature of the diatom that distinguishes itself from other types of microalgae is its silicified cell wall, also known as frustules. Diatoms’ frustule does not only provide architectural support and enhance nutrient uptake, but it can also accord mechanical resistance to withstand crushing forces by ocean grazers such as copepods^16^.

For many years, researchers have been studying the mechanism of biosilica deposition in the cell walls of diatoms. Since then, two key components, namely long-chain polyamines (LCPA) and silaffins (phosphoproteins)^13^ specifically in *Cylindrotheca fusiformis*, have been identified. In silicification, a form of biomineralization, diatoms are able to take up silicic acid available in the ocean and, through the process of polycondensation, deposit silica to reinforce their cell wall^17^. In polycondensation, LCPA acts as an acid–base catalyst. Its deprotonated amine backbone (base) accepts a proton from silicic acid, facilitating the formation of a reactive silanolate group, which can then attack the Si atom center of a neighboring silicic acid, forming a siloxane bond and release water in the process^18^. This siloxane bond confers diatoms’ strong mechanical properties, which we know today.

Since this biomineralization process occurs in diatoms, no harsh conditions were required to synthesize biosilica. Mimicking the silicification process can circumvent the need for harsh conditions (extreme temperatures or pH) in silica production, which may be energy-intensive^19^. More importantly, unlike other renowned methods such as the “Stöber” synthesis whereby surfactants cetyltrimethyl ammonium bromide (CTAB) and silica coupling agents are used in order to ensure homogenization and minimal phase separation, neither surfactants nor coupling agents are required in this process. This does not only mean a complete elimination of the need to use additional chemicals, it also simplifies the washing procedure as well, greatly reducing the environmental impact and making it a greener approach towards silica and silica composite production.

In this work, silk fibroin (SF) a protein-based biopolymer extracted from *Bombyx mori* silkworm cocoon^20^ was used to fabricate composite aerogels. Besides being available in abundance at low cost, our group have used SF for the successful mechanical reinforcement of pristine silica and silsesquioxane aerogels for different application purposes^21–24^. While amino acids containing amino functional groups in the SF chain are less common (occurring at a rate of less than 0.5%), the SF structure includes a variety of amino acids, such as tyrosine, that can be easily modified for subsequent crosslinking with amino-functionalized polymers. Therefore, in our biomimetic strategy, we modified the surface of tyrosinase-mediated oxidized SF (SFO), with polyethyleneimine (PEI), which will confer plenty of amine groups onto the SF polymer as well as confer better mechanical flexibility to SFO.

**Figure 1:**
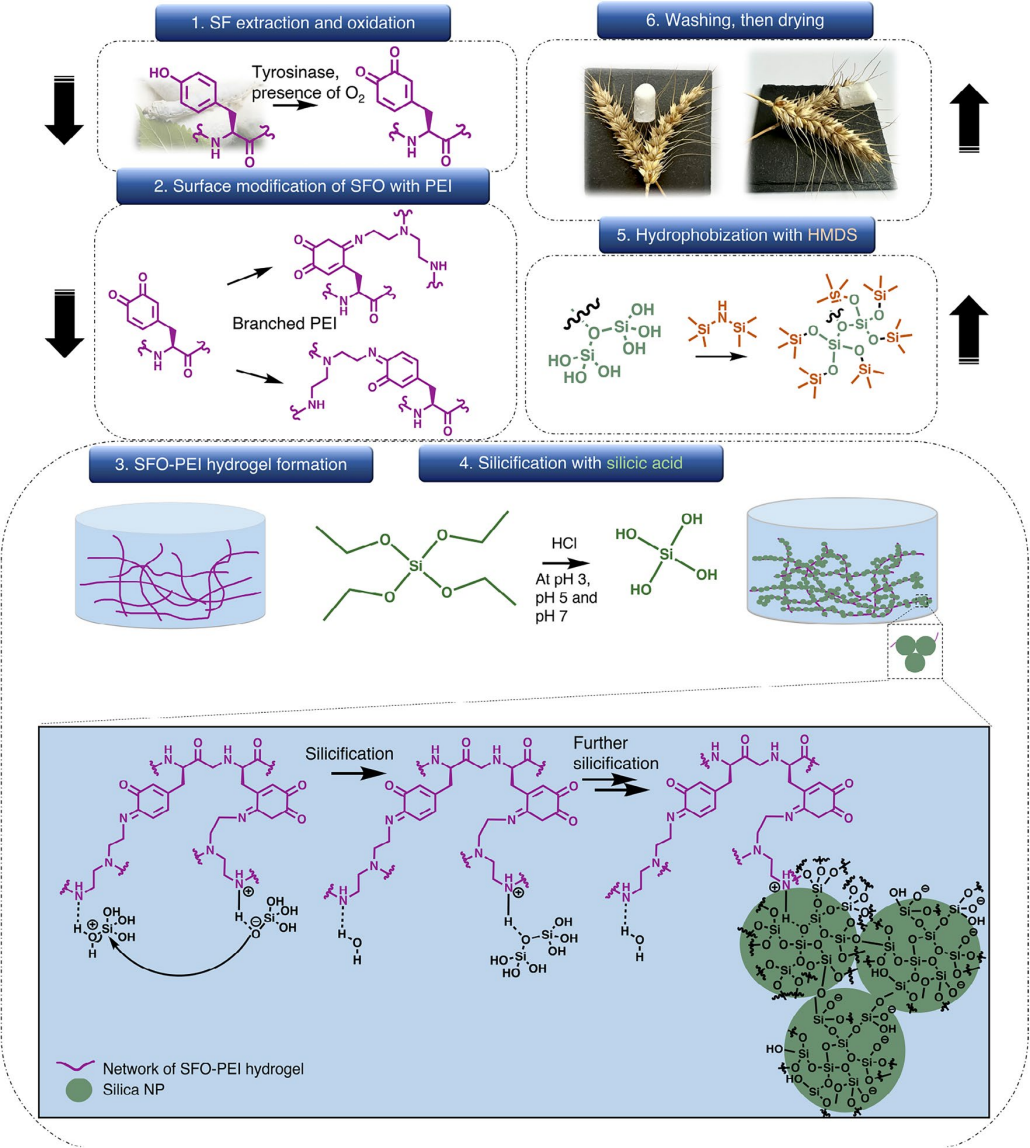
Fabrication procedure of diatom-inspired composite aerogel. (1) SF was extracted from the cocoon of *B. mori* silkworm. Tyrosine amino acid of SF was oxidized with tyrosinase enzyme in the presence of oxygen (SFO). (2) The SFO is then surface modified with branched PEI to obtain SFO-PEI. (3) The SFO-PEI-Silica hydrogel was developed. (4) TEOS was hydrolyzed by an acid catalyst to form silicic acid for silicification. (5) The hydrogel composite obtained was hydrophobized with HMDS. (6) The hydrogel was washed, and then the composite aerogels and cryogels were obtained after supercritical CO_2_, and freeze dryings, respectively.

These amine groups catalysed the silicification of SFO-PEI gel surface with silicic acid (in a similar manner as LCPA and silaffins of diatoms) to produce a globular silica network structure on the surface of the primary network of SFO-PEI matrix in an aerogel network. Here, the SFO-PEI gel matrix has a dual purpose: (1) It serves as a supportive, flexible network/scaffold for the silica network to grow in situ; (2) It provides plenty of amine functional groups as a catalyst for silicification/silica polycondensation to occur. The diatom-inspired silica-polymer aerogel composite was then hydrophobized with hexamethyldisilane (HMDS) in order to minimize shrinkage during supercritical drying. The final silica aerogel composite was mechanically reinforced with positive attributes contributed by both the underlying network/scaffold of SFO-PEI as well as the secondary silica network obtained by silicification. Figure 1 indicates the general procedure for the development of diatom-inspired silica composite aerogels.

The developed composite aerogels demonstrated superior mechanical strength compared to conventional SF-silica composite aerogels. They are both cyclically compressible and 1.4 times stronger. These observations can open doors to a much more comprehensive range of applications for silica-polymer composite aerogels, such as their use as folding and wrapping insulating materials in aerospace, building, and electronic applications^25^, which were not achievable with pristine and most aerogel composites due to their suboptimal mechanical flexibility. Additionally, since both silica and SF are biocompatible and biodegradable components^26^, the SF-silica composites aerogel can also be used as a temporary implant in regenerative medicine, where surface roughness and flexibility can augment and improve the effectiveness of cell-material interactions^27^.

## Materials and methods

### Materials

*B. mori* silkworm cocoons were obtained from Treenway Silks, USA. Tetraethylorthosilicate (98% purity; TEOS), ethanol (99.9%; EtOH), calcium chloride (99.99%; CaCl_2_), sodium carbonate (Na_2_CO_3_), polyethyleneimine (PEI) (average M_w _$\sim$ 25,000 by LS, average M_n_ $\sim$ 10,000 by GPC; branched) and tyrosinase from mushroom (lyophilized powder; $\ge$ 1000 unit/mg solid) were procured from Sigma-Aldrich. Hexamethyldisilazane (HMDS) (98% purity; (CH_3_)_3_SiN(H)Si(CH_3_)_3_) was purchased from Carbolution Chemicals GmbH. All chemicals listed were used without further purification. The SnakeSkin™ dialysis tubing with molecular weight cutoffs (MWCOs) of 3.5 kD was purchased from Thermo Fischer Scientific.

We have obtained the permissions to collect the wheat grass from local farmer. All procedures were conducted in accordance with the guidelines.

### Silk fibroin extraction

The cocoons of *B. mori* silkworm were used for the extraction of silk fibroin. The cocoons were cut into pieces, and 5 g was taken for degumming. In degumming, the cocoons were boiled in 2 L of 0.02 M Na_2_CO_3_ for 30 min to remove sericin. Then, large volumes of distilled water were used to rinse the fibers to remove impurities. After leaving the fibers to dry overnight at 40 °C in an oven, 4 g of the dried fibers were dissolved in 20 mL of Ajisawa’s reagent at 80 °C for 2 h. The Ajisawa’s reagent consists of a mixture of CaCl_2_, EtOH, and H_2_O in a ratio of 1:2:8. Dialysis of the resulting brown solution was performed against distilled water by the addition of the resulting solution into a dialysis tubing that is sealed on both ends and immersing it into a beaker with 2 L of distilled water for 48 h. The distilled water in the beaker is replaced every hour. A cloudy yellowish SF solution obtained in the dialysis tubing was centrifuged twice, each time at 9000 rpm for 30 min to obtain the supernatant. To determine the concentration of the supernatant, the weight of an empty petri dish was measured. After the addition of a small, predetermined volume of the supernatant in the petri dish, it was allowed to dry in an oven at 40 °C. Once the SF solution is dried, its weight was calculated and its concentration determined. To obtain a 4 wt% SF solution, the supernatant was further diluted with distilled water and stored at 4 °C until further use.

### Enzymatic oxidation of silk fibroin

The tyrosine in silk fibroin was selectively oxidized by adding 1 mL of 1000 units/mL tyrosinase solution to 28 mL of the SF extract. The resulting mixture was placed in an oven at 40 °C for 24 h. The oxidized SF (SFO) extract is then stored in a polypropylene (PP) container at 4°C in the fridge for later use.

### Preparation of PEI solution

Highly viscous PEI (5 g) was dissolved in distilled water (45 g) under stirring for approximately 10 min to prepare 10 wt% of PEI solution. The unmodified clear solution of pH 10 was stored at room temperature and protected from light.

### Synthesis of SFO:PEI hydrogel

To prepare SFO-PEI hydrogel, 2 g of the SFO solution was homogenized with 1 g of PEI solution. 2 mL of ethanol was added dropwise with stirring. The mixture was then degassed in a desiccator under reduced pressure for about 10 min to remove air bubbles in the mixture. Afterward, care was taken when filling the mixture in a cylindrical PP molding container (modified single-use syringes consisting of only a barrel with opening at two ends and a plunger on one end of the barrel) so that no new air bubbles were introduced. The opening of the moulding container was then sealed. The samples are stored upright and placed in the oven at 40 °C for 24 h for gelation and aging. After 24 h, the samples were demolded with the use of the plunger into a container with distilled water. Washing with distilled water was performed three times over a period of 24 h.

### Synthesis of SFO:PEI: silica composite

To prepare the composite aerogel, varying concentrations of TEOS solution were prepared (0.08–0.5 M). 0.5 M of TEOS will be used as an example. For hydrolysis, HCl (12 M) was added to the 0.5 M TEOS solution with agitation at high speed. KOH (10 M) was then added under stirring to increase the pH of the solution to pH 3. (Depending on the concentration of TEOS, the target pH varies to avoid premature gelation. For TEOS concentration below 0.5 M, the target pH is pH 5). After the last washing (explained in “Synthesis of SFO:PEI hydrogel” section), the SFO-PEI hydrogel was then immersed into the prepared TEOS solution for varying time periods (6 h and 26 h) for silicification to occur.

### Drying

Samples were dried by freeze drying and supercritical drying according to below:

- *Freeze drying (FD)* The as-prepared hydrogel in “Synthesis of SFO:PEI: silica composite” section were unidirectionally freeze-casted at a constant rate on top of a copper surface cooled with liquid nitrogen (− 196 °C) and then transferred to a freeze dryer to be dried at − 55 °C and 0.04 mbar over 2 days.
- *Supercritical drying (SCD)* Firstly, EtOH was used for solvent exchange to prepare SFO:PEI:Silica alcogel from hydrogel samples. Water was replaced by EtOH every 1–2 days with a total of five solvent exchanges over a period of 7 days. Water-free alcogel was placed in an ethanolic 10 wt% HMDS solution at room temperature for 6 h before the last EtOH solvent exchange for hydrophobization of monoliths. After the last EtOH solvent exchange, the monoliths were placed in a supercritical pressure chamber. The EtOH solvent was replaced by liquid CO_2_ with five CO_2_-extractions over a period of 2.5 days (twice daily, with 8 h intervals). This was performed by delivering gaseous CO_2_ into the chamber with pressure at about 55–57 bar, whereby liquid CO_2_ will replace EtOH as the solvent. After more than 2 h, EtOH was removed via a different valve. After solvent exchange, supercritical drying at the supercritical point of CO_2_ was performed.

### Characterization techniques

Bulk density ($\rho$) is calculated by Eq. (1) below with the mass ($m$) and volume ($V$) of the cylindrical aerogel, where V is obtained from the height ($h$) and diameter ($d$) of the cylindrical aerogel:

1 $$\rho =\frac{m}{V}=\frac{4m}{\pi {d}^{2}h}.$$

UV–Vis spectrum was recorded in the range of 250 to 800 nm with a wavelength spacing of 1 nm on a Lamda 950 (PerkinElmer) Spectrophotometer at room temperature. Scanning Electron Microscopy (SEM) images were obtained with a Zeiss ULTRA Plus SEM at 10 kV with an in-lens detector and working distance of around 3 mm. Energy-dispersive X-ray (EDX) spectra were obtained in the SEM using an X-Max silicon drift detector. Attenuated Total Reflectance-Fourier Transform Infrared (ATR-FTIR) spectroscopy was obtained with a Brucker Vertex 70 spectrometer with a 4 cm^−1^ resolution, with a scan range from 400 to 4000 cm^−1^. Thermogravimetric analysis was obtained from a TGA 4000 thermogravimetric analyzer (PerkinElmer) with a heating rate of 10 °C/min, from 30 to 900 °C under a nitrogen flow of 20 mL/min. Mechanical characterization on monolithic cylindrical samples was performed at 25 °C using a DMA 3200 universal mechanical testing machine (TA Instruments) with a force output of up to 500 N. Stress–strain curves were plotted in compression mode, and Young’s modulus values were calculated from the linear range of the curves occurring at $5.81\pm$ $2.1$%. A constant compression rate of 0.4 mm/s was used across 3 different types of measurements: (a) compression to 80% strain, (b) dynamic loading–unloading cycle (8 cycles from 0 to 80% strain with an incremental strain of 10% for each cycle) with 0.2 mm/s for unloading and (c) static loading–unloading cycle (10 cycles at 20% strain for each cycle) with 0.1 mm/s for unloading.

## Results and discussion

In this work, we use a simple, straightforward, and green technique (as illustrated and described in Fig. 1) to develop SF-silica composite aerogels with improved mechanical strength and flexibility, and minimal compromises to their bulk density. This is attributable to the method of reinforcement in this work, where silica nanoparticle formation is able to extend from the surface of the SFO-PEI primary network via a simple silicification process inspired by nature. Here, we have discussed the optimization of the synthesis parameters (TEOS concentration, pH, hydrophobization) and drying methods in order to accord stable monoliths.

### Chemical properties

**Figure 2:**
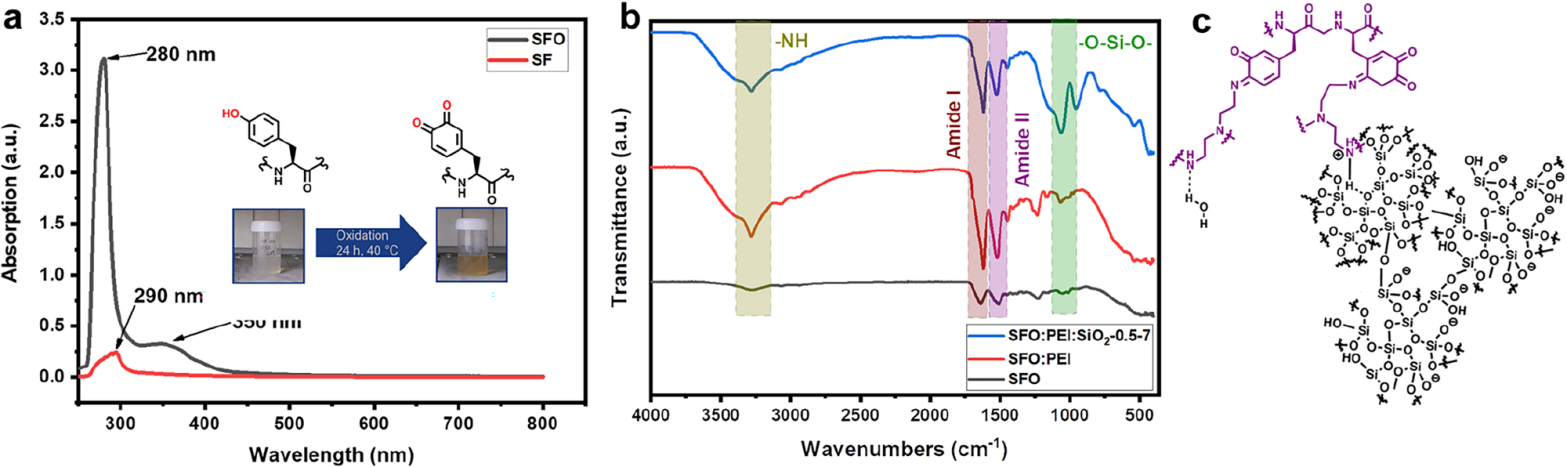
(**a**) UV–Vis spectrum of SF and oxidized SFO. (**b**) ATR-FTIR spectra of SFO, SFO:PEI and SFO:PEI:SiO_2_-0.5–7. (**c**) Chemical structure of silicified SFO:PEI composite.

The oxidation of SF to SFO is accompanied by a color change from white to yellow. In the UV–vis spectrum of Fig. 2a, a strong absorption peak at 280 nm which is attributable to *o*-quinones^28^ in the SFO can be observed. There is also a shoulder peak at 350 nm which may originate from the reaction between the *o*-quinones and amine groups found on the side chain of amino acids in silk fibroin^29^, such as Arginine and Lysine. In the ATR-FTIR spectra in Fig. 2b, subsequent synthesis of SFO to accord the SF-silica composite is monitored. When PEI is added to SFO, there is an observable N–H peak at 3281 cm^−1^. After silicification, a prominent siloxane peak at 1062 cm^−1^ is also observed, indicating the presence of silica deposited on the SFO:PEI hydrogel network. This is illustrated in Fig. 2c with silica nanoparticles (black) bonded to the SFO-PEI network (purple) after the silicification process.

### Physical and mechanical properties

Comparing the densities of the monoliths obtained from the two different drying techniques (freeze drying and supercritical drying) shows that cryogels of lower bulk densities (between 0.035 ± 0.001 g/cm^3^ and 0.048 ± 0.009 g/cm^3^) were obtained from freeze drying compared to aerogels of supercritical drying (between 0.079 ± 0.007 g/cm^3^ and 0.186 ± 0.003 g/cm^3^)—See Table 1. On closer inspection, the aerogels that were hydrophobized had low densities of 0.079 ± 0.007 g/cm^3^ compared to non-hydrophobized aerogels of 0.186 ± 0.003 g/cm^3^. This was in the same order of magnitude as those obtained from freeze drying. This demonstrates the effectiveness of hydrophobization in minimizing the collapse of pores in the hydrogel, as well as preventing sustained polymerization, as hydrophobized aerogels have lower densities compared to their non-hydrophobized counterparts.

**Table 1:** Physical properties of cryogels and aerogels synthesized in this study.

Cryogel/aerogel	TEOS concentration [M]	pH	Hydrophobization	Bulk density, ${\rho }_{bulk}$ [g/cm^3^]	Volume shrinkage, $\eta$ [%]
Freeze drying (FD)					
SFO:PEI	–	–	–	$0.037\pm 0.003$	$8.6\pm 4.5$
SFO:SiO_2_-0.08–3	0.08	3	–	$0.035\pm 0.001$	$0$
SFO:PEI:SiO_2_-0.08–3	0.08	3	–	$0.048\pm 0.009$	$13.1\pm 2.9$
Supercritical drying (SCD)					
SFO:PEI	–	–	–	$0.124\pm 0.020$	$71.7\pm 3.2$
SFO:PEI:SiO_2_-0.5–1	0.5	1	–	$0.186\pm 0.003$	$76.5\pm 1.8$
SFO:PEI:SiO_2_-0.5–1*	0.5	1	Yes	$0.079\pm 0.007$	$40.0\pm 8.2$

The samples are named as SFO:PEI:SiO_2_-x–y (where x and y refers to the concentration of TEOS solution and pH of TEOS solution respectively).

*Hydrophobization performed.

The high bulk densities of silicified aerogels obtained from supercritical drying may be attributed to the higher monomer content of TEOS (0.5 M in SCD aerogels compared to 0.08 M in FD cryogels). There is a correlation between TEOS content and the pore volume of the gels formed. When TEOS content increases, there is a decrease in the average pore volume of the aerogels formed^30^. This leads to an increase in the bulk density of the aerogels.

Besides monomer content, the drying method may also explain the higher bulk densities of aerogels obtained from SCD.

In SCD, the solvent exchange step is inherently time-consuming. While performing the solvent exchange to prepare the alcogels for supercritical drying, siloxane groups in the network may continue to be produced via polycondensation of silanol groups of the silica nanoparticles. This reduces the porosity and ultimately increases the density of the resulting aerogel. This sustained polymerization effect is less pronounced in hydrophobized aerogels.

Additionally, the depressurization rate during SCD can be better optimized according to the size of the monolith. If the depressurization rate is too fast, the pressure within the gel network may build up as the supercritical fluid within the network cannot escape fast enough, resulting in a higher pressure compared to that outside of the network^31^. This, contrary to our expectation of SCD, can cause cracking in the monolith, even though the monolith remains intact^32^.

**Figure 3:**
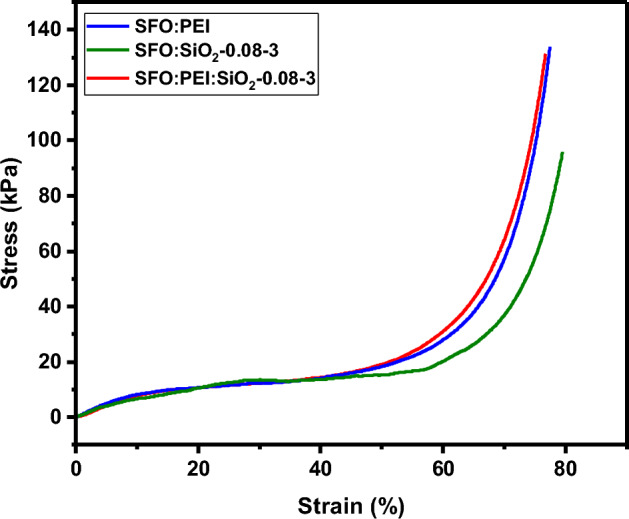
Stress–strain curve of SFO: PEI, SFO: SiO_2_-0.08–3 and SFO:PEI:SiO_2_-0.08–3 cryogels.

From Fig. 3, compressive tests performed show that the cryogels were able to withstand up to 80% strain without failure. The linear, plateau, and densification regions can also be identified^33^ from the curve. Compressive behaviour such as compressive strength, Young’s modulus, and yield strength of individual cryogels could be evaluated. The young’s modulus of 0.873 kPa is the smallest for the SFO:PEI:SiO_2_-0.08–3 cryogel, indicating that this composition of cryogel is the most flexible out of the 3 different types presented in Table 2. This shows that even with silicification, the flexibility accorded by the SF to the silica inorganic network is not compromised but also retained. At the highest yield strength of 3.37 kPa calculated at 2% offset stress, it is also able to withstand elastic deformation the best compared to the other compositions of cryogels. The compressive strength was also improved by 1.4 times compared to SFO:SiO_2_-0.08–3 cryogel. This suggests that silicification with involving amino groups in PEI provides an advantage over silicification without PEI. In PEI, the amino groups are appropriately positioned, providing a cooperative action which enabled the condensation of two silicic acid molecules as shown in Fig. 1. This resulted in more controlled interactions between SFO and silica. Unlike less controlled interactions, which may undermine the overall mechanical properties caused probably by encapsulation of the silica nanoparticle in the aerogel.

**Table 2:** Mechanical properties of cryogels synthesized in this study.

Cryogel	Compressive strength, ${\delta }_{max}$ [kPa]	Young’s modulus, ${\rm E}$ [kPa]	Yield strength, $\sigma$ [kPa]
Freeze drying			
SFO:PEI	132.97	$1.052$	$1.81$
SFO:SiO_2_-0.08–3	95.69	$0.909$	$2.00$
SFO:PEI:SiO_2_-0.08–3	131.76	$0.873$	$3.37$

The samples are named as SFO:PEI:SiO_2_-x–y (where x and y refers to the concentration of TEOS and pH, respectively).

Cryogels of the following composition: SFO:PEI, SFO:SiO_2_-0.08–3 and SFO:PEI:SiO_2_-0.08–3 were subjected to more detailed mechanical tests such as dynamic and static loading–unloading cycles.

**Figure 4:**
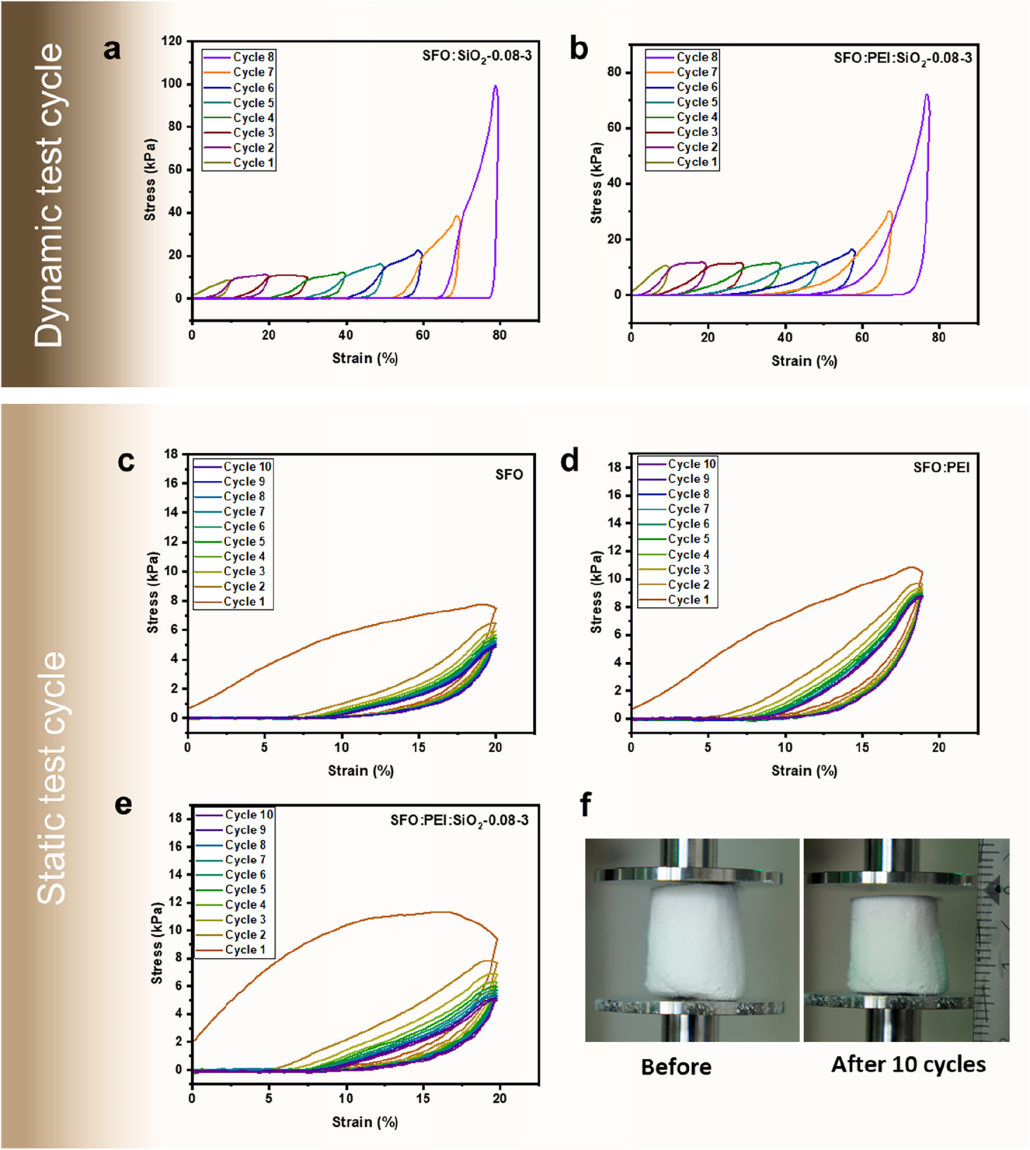
(**a,b**) Compressive tests, including dynamic test cycles up to 80% strain and (**c–e**) static test cycles up to 20% strain, performed on cryogels (**f**) Photographs of SFO:PEI composite before and after static test cycle.

In dynamic loading–unloading cycle tests, the stress–strain curves in Fig. 4a,b shows that all cryogel samples generally undergo negligible strain recovery from 10% strain amplitude, as the strain does not return to zero when the load is removed. This means that the cryogels experience a permanent residual deformation above 10% strain. The hysteresis curves in Fig. 4a,b also show incremental small slopes at low strain amplitudes and a drastic increase at high strain amplitudes. The hysteresis curve gives an insight into the energy dissipation during deformation. For Fig. 4b, the hysteresis curve shows a low and constant energy dissipation up until about 60% strain, and beyond 60% strain, the energy dissipation increases drastically. This is in contrast to Fig. 4a, whereby the energy dissipation increases at every incremental strain up to 60% and after that drastically. Energy dissipation can be explained by the microstructure of the cryogel as well as its deformation mechanism^34^. The observation in Fig. 4b may be attributed to the physical crosslinking between the silica nanoparticle and the SFO biopolymeric network. From the controlled silicification in SFO:PEI:SiO_2_-0.08–3, homogenous distribution of the silica nanoparticle on the SF biopolymer can be encouraged, allowing the entire network to slide and dissipate energy when stress is applied.

The static loading–unloading cycle tests, Fig. 4c–f show that, in general, all cryogel samples underwent cyclic softening at constant cyclic strain amplitude (20% strain). The hysteresis loops during loading and unloading indicate the occurrence of cyclic softening. Cyclic softening is a common occurrence whereby the cyclic stress amplitude decreases most significantly in the response to repeated compression-decompression cycles in the same strain in the first few cycles, and its effect becomes less significant in subsequent cycles^35^. Cyclic softening is more pronounced in SFO:PEI compared to SFO and its silicified counterpart SFO:PEI:SiO_2_-0.08–3. This suggests that silicification preserves the ability of SFO to provide fatigue resistance, and does not undermine the cyclic softening effect.

### Microstructure and composition

**Figure 5:**
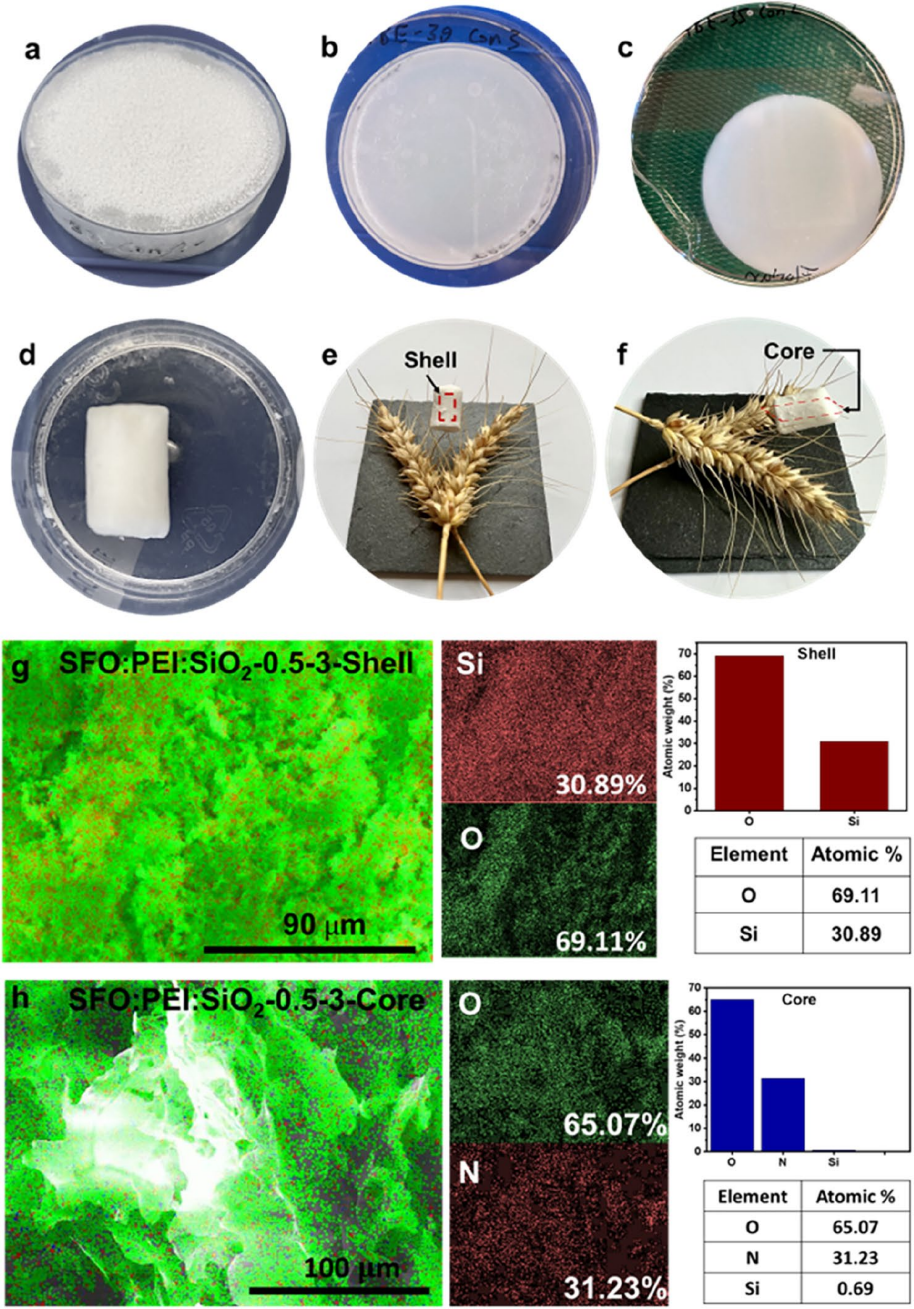
(**a,b**) SFO: PEI hydrogel samples in a petri dish (50 mm diameter, 9 mm height) before demolding. (**c**) Demoulded SFO:PEI hydrogel sample. (**d**) SFO:PEI:SiO_2_ cylindrical aerogel sample (9 mm diameter, 18 mm height). (**e,f**) Top and lateral view of composite aerogel monolith on top of wheat grass indicating shell and core. (**g**) SEM–EDX elemental mapping of SFO: PEI:SiO_2_-0.5–3-Shell and (h) SFO:PEI:SiO_2_-0.5–3-core.

Figure 5a–f indicates the digital photo of the monolith SFO-PEI and SFO-PEI:SiO_2_ hydrogel and aerogel samples in different size and macroscopic forms. Even though sample macroscopically are homogeneous with ample structural integrity, microscopically, the exterior (shell) parts are more silicified than interior (core) parts.

Scanning Electron Microscopy-Energy Dispersion X-ray (SEM–EDX) images can shed light on the macrostructure and composition of the aerogel. The monoliths fabricated were observed in Fig. S1 to have a hard shell structure, which upon mechanical stress exposes a dense core. SEM–EDX images of the shell and core were taken and compared.

From Fig. 5g,h, SEM–EDX analysis shows that silicification key elements of O and Si are present in the monolith. With an Si atomic percentage of 30.89% in the shell, one can infer that Si is homogenously distributed on the SF biopolymeric network. However, at the core, the number of Si atoms were negligible with an atomic percentage of only 0.69%. This shows that compared to the core, silicification mostly occurred on the shell of the SFO:PEI aerogel.

**Figure 6:**
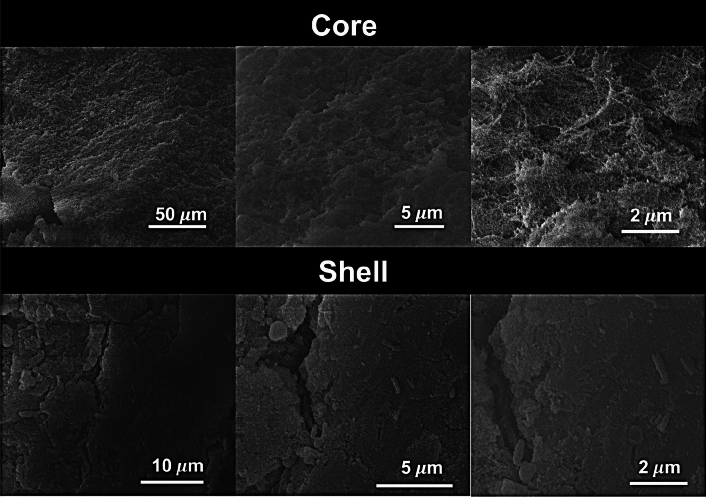
SEM images obtained from supercritically dried aerogel with composition SFO:PEI:SiO_2_-0.5–3* (*Hydrophobization performed).

Comparing the core and shell SEM images of SFO:PEI:SiO_2_-0.5–3* in Fig. 6, one can see that the microstructure of the core differed greatly from that of the shell. At first glance, the core shows surface roughness. On higher magnification of the core, fibers which are characteristic of SF network (SFO:PEI) become visible. This observation is also in agreement with the EDX analysis in Fig. 6, with little to no silicification occurring in the core, the surface-modified SF remained as a biopolymeric network within the core. The shell, on the other hand, showed a much flatter surface with visible cracklines.

**Figure 7:**
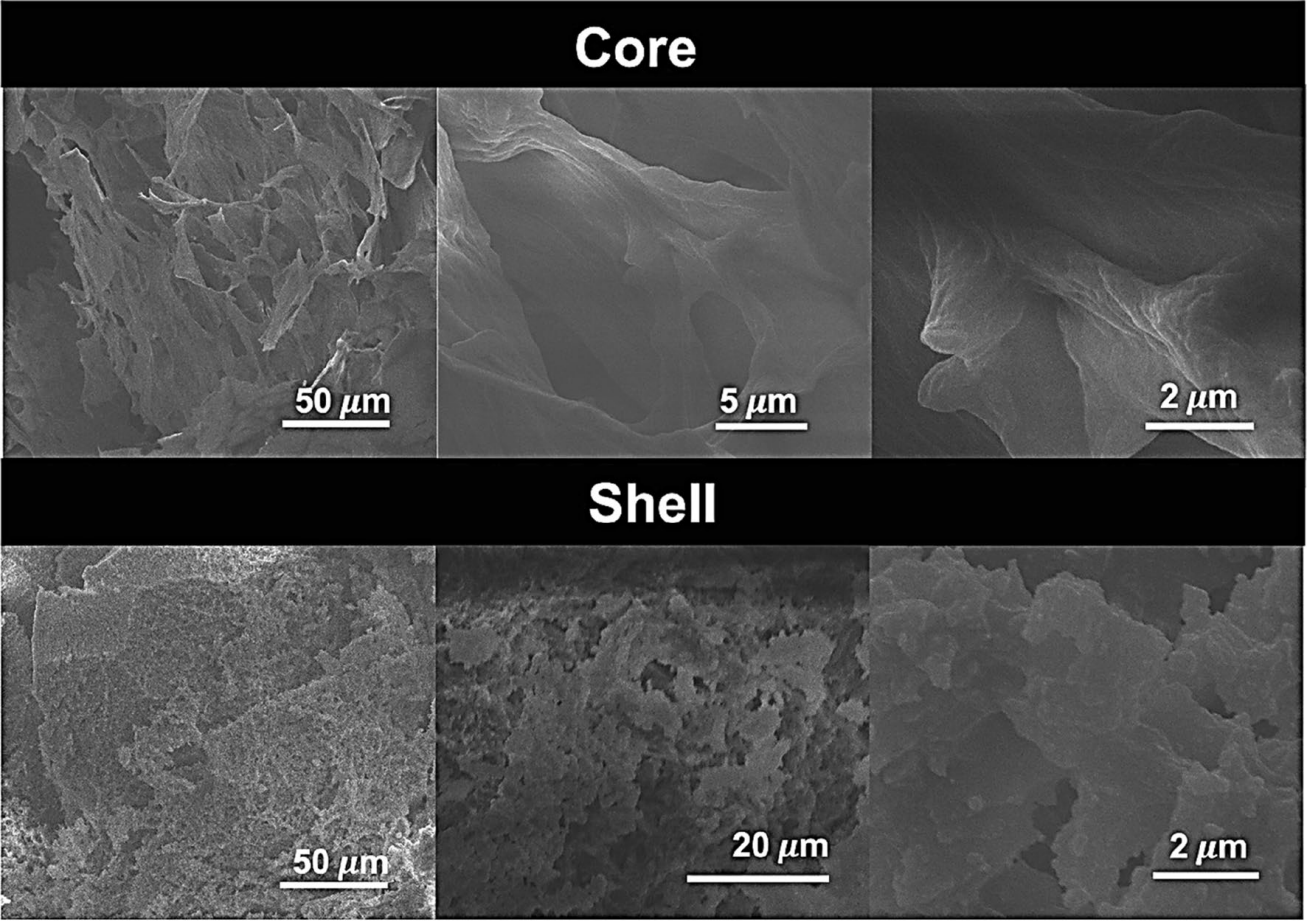
SEM micrographs of SFO:PEI:SiO_2_-0.5–3 cryogels.

Figure 7 shows the SEM images obtained from the same monolith (SFO:PEI:SiO_2_-0.5–3) presented in Fig. 5. Between the core and shell, the core is much smoother and fibrous compared to the shell which shows surface roughness. The fibrous microstructure of the core, is similar to what was presented in Fig. 6, and attributed to the silk fibroin network (SFO:PEI). What stands out differently is the 3D structure of the cryogel samples core in Fig. 7 as compared to that of the supercritically dried core in Fig. 6. This implies that aerogels experienced pore collapse and lose of their 3D structure which was prominent in cryogels. This results in a greater bulk density of aerogel compared to cryogel samples. The SEM image of the shell of cryogel displays surface roughness, which can be attributed to the deposition of the silica nanoparticles as a result of silicification. Clusters of dense areas may represent the aggregation of silica nanoparticles as a result of polycondensation.

Comparing the cracklines on the shell between hydrophobized and non-hydrophobized monoliths in Fig. S2 shows the effectiveness of hydrophobization. Hydrophobization can impede continued silicification as the hydroxyl groups of the silica nanoparticles are methylated. The presence of methylated silica nanoparticles can also prevent the formation of cracks during supercritical drying. From Fig. S2, the monolith, which was not hydrophobized shows marginally more cracklines compared with the monolith which was hydrophobized. Since this difference was hardly noticeable, it can be proposed that crack formation was not minimized to a large extent in hydrophobization. On the other hand, from the bulk densities of hydrophobized and non-hydrophobized monoliths, one can say that continued silicification was reduced to a significant extent with hydrophobized monoliths. This is reflected in the lower density of hydrophobized monolith (0.079 $\pm$ 0.007 g/cm^3^) compared to that of the non-hydrophobized monolith (0.186 $\pm$ 0.003 g/cm^3^).

### Chemical structure characterization: influence of synthesis parameters

ATR-FTIR analysis provides more specific information by identifying the type of bonds present in the fabricated SF-silica composites. Considering the difference between the composition of silica in the core and shell of the aerogels, the effect of pH, concentration of TEOS, and hydrophobization will be discussed.

**Figure 8:**
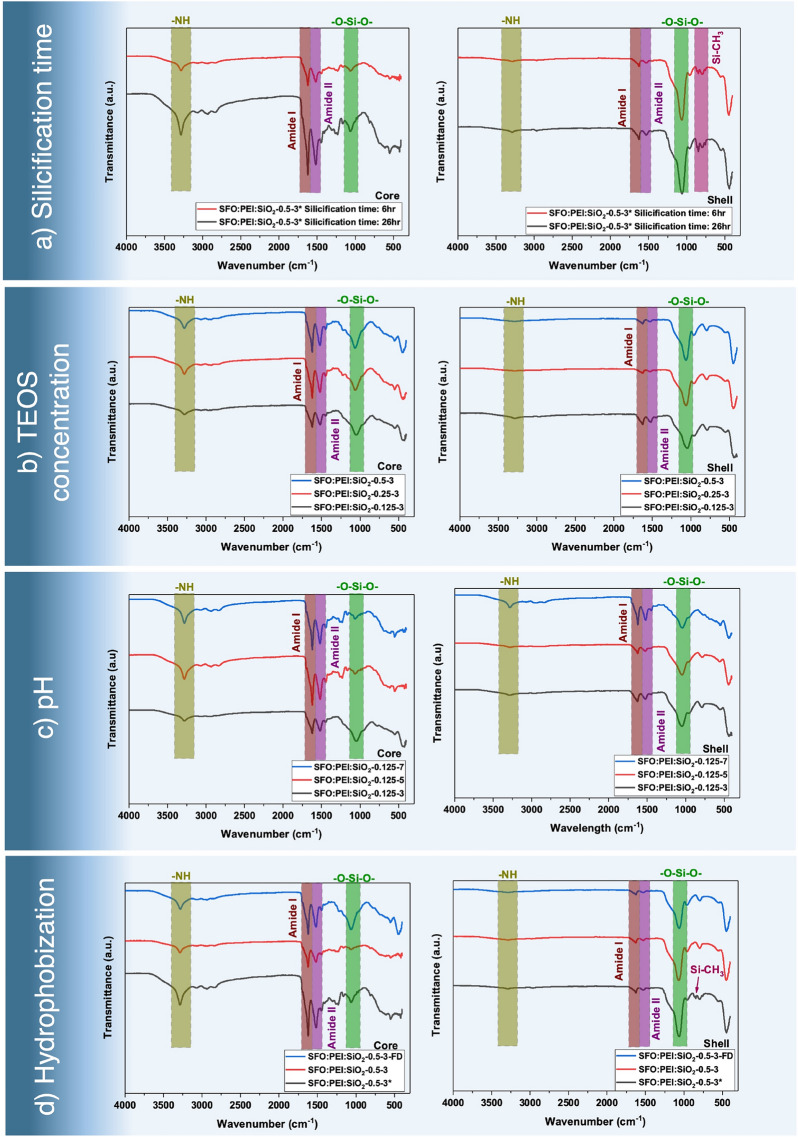
ATR-FTIR spectrum of core (left) and shell (right) of composites prepared with (**a**) varying silicification time, (**b**) varying TEOS concentration, (**c**) varying pH values, and (**d**) hydrophobization.

ATR-FTIR is a popular method and is widely used to elucidate secondary protein structure^36^ in SF-based materials. As seen from Fig. 8, the characteristic bands of SF are the amide bands, namely Amide I, Amide II, and Amide III bands. Amide I bands (between 1600 and 1700 cm^−1^) correspond to the C=O stretching vibration. Amide II bands (between 1500 and 1600 cm^−1^) correspond to the C–N stretching vibration combined with N–H bending. Amide III bands (between 1175 and 1310 cm^−1^) have very low intensity and correspond to C–N stretching vibration in combination with N–H bending vibration, C–C stretching, and C–H bending. It is worth mentioning that all amide bands are conformationally sensitive, with Amide I being more sensitive than Amide II^37^.

In addition to the amide bonds, we are also interested in the siloxane and silanol bonds, which show up as bands at 1000 to 1130 cm^−1^ and 810 to 950 cm^−1^, respectively. At about 3300 cm^−1^ is the N–H stretch, which is not dependent on the amide backbone but is influenced by the strength of the hydrogen bonding of the amino groups of the PEI.

The time required for silicification has been established to be largely completed in 6 h. From Fig. 8a, the intensity of the siloxane bond at about 1100 cm^−1^ for composites silicified for 6 h and 26 h did not differ significantly, especially in the shell. The effect of hydrophobization was also visible. In the shell of the hydrophobized composites, two additional peaks at about 750 cm^−1^ and 840 cm^−1^, corresponding to the vibration of polysiloxanes^38^ (Si–CH_3_) were also present.

One of the most striking observations in Fig. 8a–c between the core and the shell of the SFO:PEI:Silica composites is the intensity of the peak corresponding to the siloxane bond. In general, the siloxane peak is significantly more intense in the spectrums obtained from the shell of the composites compared to those obtained from the core of the same composites. This is in agreement with the SEM–EDX analysis shown in Fig. 5g,h which reflects the greater degree of silicification and sol–gel reaction in the shell compared to the core, leading to a greater Si elemental composition in the shell compared to the core.

Due to the variation in the degree of silicification and sol–gel polymerization in the core and shell of the SFO:PEI:Silica composite, the core and shell may adopt different secondary conformations. Analysis of the frequency and intensity of the Amide I and Amide II peaks can provide more insight into their secondary conformation. The Amide I and Amide II peaks have significantly larger intensities and are very slightly red-shifted to smaller wavenumbers in the core compared to the shell of the composites. For example, in Fig. 8c, the Amide I and II peak of SFO:PEI:SiO_2_-0.125–5 is 1617 cm^−1^ and 1517 cm^−1^ respectively, at the core and 1625 cm^−1^ and 1528 cm^−1^ respectively, at the shell. This may suggest that the core has retained its $\beta$ sheet conformation while the shell may have lost, to some extent, some of its $\beta$ sheet conformation during silicification and polymerization and formed random coils in the composites. The strength of the hydrogen bonding may have been weakened in the shell, showing up as a less intense Amide I and Amide II peak.

#### Effect of TEOS concentration

The concentration of the TEOS used in this work varies from 0.125 to 0.5 M. Since the intensity of the siloxane peak remains similar mainly in Fig. 8b, there is no notable effect of increasing TEOS concentration within this range. It is plausible that the TEOS concentration range may be too narrow and too low to see a substantial effect on silicification in Fig. 8b.

While there is little effect on silicification with varying TEOS concentration from 0.125 to 0.5 M, an effect on the secondary conformation of the SFO:PEI:Silica composites, particularly in the core, was observed. In Fig. 8b, we see that as the TEOS concentration increases, the intensity of the Amide I and Amide II peak in the core increases as well. This shows that there is stronger hydrogen bonding of the amide backbone with increasing TEOS concentration. This is a reasonable observation as increasing TEOS concentration increases the number of silica nanoparticles that can hydrogen bond to the amide backbone. Besides forming hydrogen bonds to the amide backbone, silicic acid can also form silica nanoparticles of different sizes and uniformity based on different degrees of hydrolysis and silicification. A higher TEOS concentration increases the condensation state of silicic acid molecules by providing more silicic acid molecules with which silicification can occur. This results in a greater silica nanoparticle size, which can affect the secondary conformation of the composite. With more extensive investigations, we can quantify the size and uniformity of these silica nanoparticles formed from different TEOS concentrations in order to shed more light on the secondary conformation of these composites.

#### Effect of pH on silicification

At the reaction pH between pH 3–7, it is noteworthy to mention that the deprotenation of silicic acid to form silanolate ions is very low^39^. Therefore, the rate of polymerization via a nucleophilic attack of a deprotonated silicic acid molecule on another silicic acid molecule is very slow.

In acidic conditions, hydrolysis of TEOS to form silicic acid is promoted. With its long chain amino groups, SFO:PEI brings two silanol groups close together by acting as an acid–base catalyst to speed up the silicification process. Since the rate of silicification is aided by the presence of long-chain amino groups on SFO:PEI, the limiting factor becomes the rate of hydrolysis and formation of the silanol groups. In a more acidic pH (pH 3), TEOS readily form silanol groups via acidic hydrolysis, which are available for silicification and condensation. Therefore, at pH 3, there is more substantial siloxane peak compared to the silicification performed in less acidic conditions. This effect is much more pronounced in the core as shown in Fig. 8c.

#### Effect of hydrophobization

From Fig. 8d, comparing the shell of the SFO:PEI:Silica composites, the polysiloxane bond (Si–CH_3_) previously observed in the hydrophobized composite was not present in the other non-hydrophobized composites. This effect is more pronounced in the shell than the core as the shell is much more silicified than the core.

The intensity of the siloxane (–O–Si–O–) bond is largely similar across all composites in the shell, showing that the hydrophobization step did not interfere with the silicification process as silicification has already been largely completed. However, in the core, it is observed that the intensity of the siloxane peak is much more intense for cryogel composites than aerogel composites. Since silicification time is not the cause, as all composites have been silicified for at least 6 h, it may be possible that aging temperature may be an influential factor on silicification in the core of the composites. The hydrogel of the cryogel composites was prepared at an aging temperature of 23 °C while the aerogel composites was prepared at an aging temperature of 40 °C.

**Figure 9:**
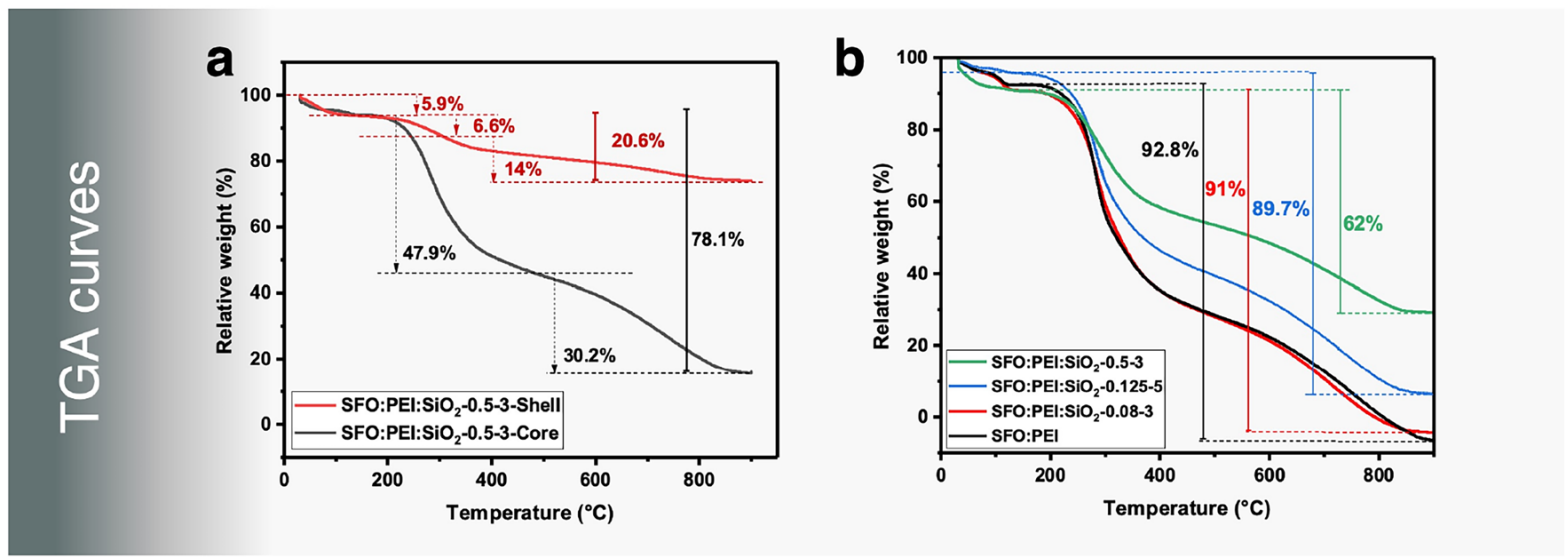
(**a**) TGA curves of SFO:PEI:SiO_2_ composite comprising of core and shell. (**b**) TGA curves of cryogel composites.

The TGA curves of the composites in Fig. 9 all show a similar trend with an initial small dip at 100 °C, one significant dip beyond 220 °C and a continuous decline between 400 and 860 °C.

The initial dip in the weight loss at about 100 °C is attributed to the evaporation of adsorbed water in the composites.

At the onset of decomposition at 220 °C, a large, significant dip in the weight loss corresponded to thermal degradation of the Silk I and Silk II structure of silk fibroin, alongside its peptide bond and the side chain of its amino acid constituents. At 400 °C is the thermal degradation onset for PEI and free silanol groups via dehydroxylation. Above 860 °C, a plateau is observed, and the remaining weight is attributed to thermally stable inorganic silica.

From Fig. 9a, as one would expect, the TGA curves of the shell of the SFO:Silica composite showed a higher weight percentage that remains compared to the core. The remaining weight percentage of the shell of the composite at about 73.5% compared to 16% of the core confirms that the degree of silicification and polycondensation in the shell is more than 4 times larger than in the core of the composite. Figure 9b shows that a higher TEOS concentration and acidic pH conditions (pH 3) leads to greater silicification and polymerization to form silica. This indicates that there is still the presence of unprotonated amino groups on the surface-modified SFO, which can serve as available binding sites for silicification and polymerization to occur. A high TEOS concentration does not only provide the precursor required, but coupled with a high pH condition, the hydrolysis of TEOS to form silicic acid for silicification is accelerated.

On the other hand, a TEOS concentration of as low as 0.08 M shows a very similar pattern compared to non-silicified SFO:PEI samples, which convert completely into the gas phase leaving no residue. This can be due to the negligible to zero silicification due to very low TEOS concentration.

An increase in TEOS concentration from 0.13 to 0.5 M (3.5 times) increases the silica residue percentage from 6.5 to 30% (4.6 times). This is a rather proportional increase, which shows that the degree of incorporation of silicic acid via silicification and polymerization is the same regardless of the starting TEOS concentration.

## Conclusion

Flexible silica composite aerogels with improved mechanical strength were fabricated inspired by the silicification process in diatoms. This strategy involves surface modification of oxidized silk fibroin with polyethyleneimine (SFO-PEI) to create long- chain polyamines resembling the function of LCPA in diatoms that are capable of acting as an acid–base catalyst by facilitating the formation of strong siloxane bonds from TEOS via silicification. The hydrogels obtained were then dried via supercritical CO_2_ and freeze-drying. Mechanical characterization techniques such as compressive strength analysis found that compared to conventional SF-silica composite aerogel, SFO:PEI-silica composite aerogels prepared via silicification was 1.4 times stronger with cyclic compressability. In terms of surface morphology and composition, SEM–EDX showed that homogenous silicification largely occurred on the shell of the composite aerogel and that freeze drying retained the 3D structure of the aerogels much better. These cryogels have also been evaluated to have lower bulk densities compared to their supercritically dried counterparts. The effect of pH, TEOS were investigated. Formation of siloxane bonds is promoted with more acidic pH conditions. While TEOS concentration did not show any significant difference in the intensity of the siloxane bonds and extent of silicification, it can have a strong effect on the secondary structure of the SF composites.

This silicification process inspired by nature relies solely on the interaction between long-chain polyamines and silicic acid under acidic conditions, presenting an advancement of the biomimetic approach toward the creation of flexible composites with mechanical strength. For a start, this process is sustainable, cost-effective and eliminates the use of toxic starting materials, which tackles key issues addressed when considering biomedical applications of biopolymeric aerogels^40^. With diffusion limitations of silane precursors into the depths of a monolith, the approach presented here is best suited for silicifying thin films or microparticles. The introduction of surface nanoroughness on these substrates, while keeping its mechanical strength, can lead to an enhancement of cell-biomaterial interactions, making these composites useful as biomedical implants, in cell attachment or drug delivery. With unique bulk and surface properties as well as biocompatibility, biopolymeric aerogels continue to hold great promise in the biomedical sector.

### Supplementary Information

Supplementary Figures.

## Data Availability

The datasets generated during and/or analyzed during the current study are available from the corresponding author on reasonable request.
