# Supplementary material for: Diatom-inspired silicification process for development of green flexible silica composite aerogels
Source: Sci Rep. 2024 Mar 23;14:6973. doi: 10.1038/s41598-024-57257-x (PMC10960801; doi:10.1038/s41598-024-57257-x)
Supplement: Supplementary file 1 — Supplementary Figures. [file 41598_2024_57257_MOESM1_ESM.docx]

Supporting Information

**Diatom-Inspired Silicification Process for Development of Green Flexible Silica Composite Aerogels**

Valerie Tan^1^, Florian Berg^1^, Hajar Maleki^1,2, *^

^1^Department of Chemistry, Institute of Inorganic Chemistry, University of Cologne, 50939 Cologne, Germany

^2^Center for Molecular Medicine Cologne, CMMC Research Center, Robert-Koch-Str. 21, 50931 Cologne, Germany

*Email: h.maleki@uni-koeln.de


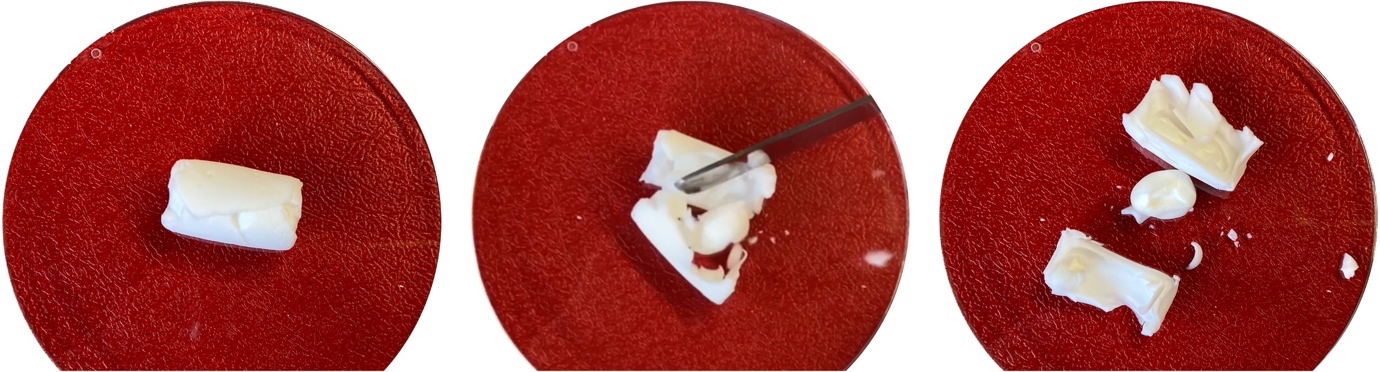


Figure S1: Photograph of SFO:PEI:SiO_2_-0.5-3 which exposes a core structure upon breakage with a spatula


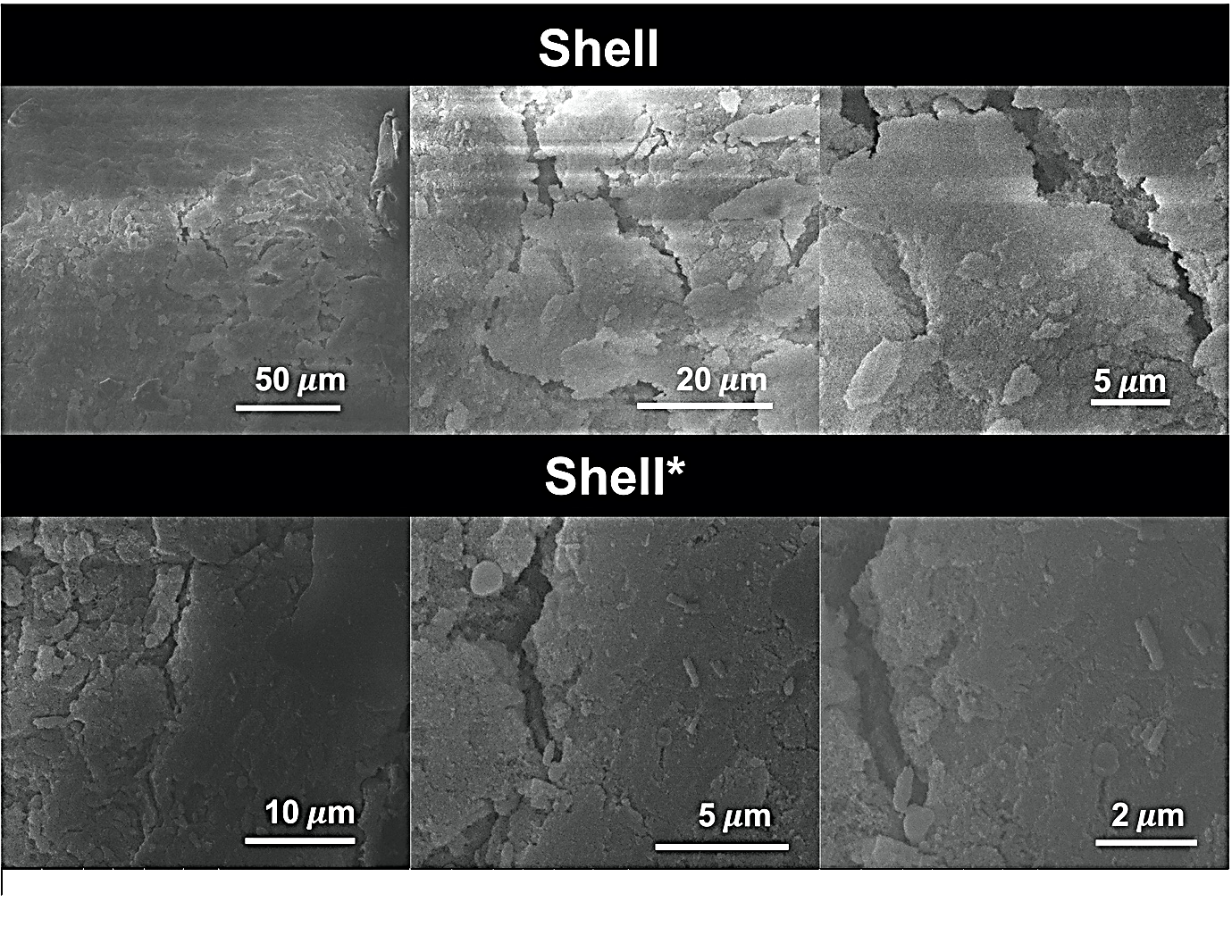


Figure S2: SEM images obtained from shell of supercritically dried aerogels with composition SFO:PEI:SiO_2_-0.5-3* and SFO:PEI:SiO_2_-0.5-3 (*Hydrophobization performed)
